# Animal Chemistry, or Chemistry in Its Applications to Physiology and Pathology

**Published:** 1847-01-01

**Authors:** 


I. Animal Chemistry, or Chemistry in its Applications to Physiology and Pathology. By Baron *Liebig*. Edited from the Author's Manuscript, by *W. Gregory*, M.D. F.R.S.E. Third Edition, revised and greatly enlarged. Part I. Octavo, pp. 256. Taylor and Walton, 1846.

II. Experimental Researches on the Food of Animals, and the Fattening of Cattle: with Remarks on the Food of Man. By *Robert Dundas Thomson*, M.D. 12mo, pp. 200. Longman, 1846.

The publications of Baron Liebig have constituted an æra in chemical science. Long a laborious cultivator in this field of research, his investigations were little known, even to the professional public of this country, until the Reports he produced at the instigation of that valuable institution, "The British Association for the Advancement of Science," disclosed him as a great practical philosopher and admirable genius. He is now the spoken-of of all men, even to the fathering upon him views and doctrines he never entertained. That his reputation is soundly based and will prove durable we can have no doubt; for, although some of his views are by no means so original as his most ardent admirers would have us believe, (and here we cannot but protest against the non-acknowledgment of Dr. Prout's priority in respect to certain of these,) and some are very hypothetical; yet, in the firm establishment of others of immense importance, in his expanded idea of chemical science, in his happy generalizations and lucid descriptions, in the light he has thrown upon various departments of physiology, and the remarkable impulse he has communicated to agricultural improvement and chemical investigation, Liebig has acquitted himself with a rare ability, and has conferred lasting benefit upon mankind.

The present part of the new edition of the "Animal Chemistry" is indeed an excellent indication of the claims of its author; containing, as it does, so many exemplifications of the correctness of the views already advanced by him, and so little in qualification of them. It is, in fact, so extended by the new matter illustrative of "the Chemical Process of Respiration and Nutrition," and by the addition of an entirely new section upon the method in which the investigation of the "Metamorphoses of Animal Tissues" should be pursued, that it may, as the Editor justly remarks, be almost considered as a new work. The general views of the author upon Respiration and Nutrition are too familiarly known to our readers to admit of our adverting to them in this notice, otherwise than by citing one or two of the additional illustrations which he brings forward; and we shall chiefly confine our attention to the new section, which indeed occupies nearly a hundred pages of the present part. Our first extract shall be taken from an interesting sub-section upon the origin and uses of the Bile.

"The taking up of dissolved foreign matters into the blood, or the absorption of such matters, is a chemico-mechanical act, which, as we have seen, extends to liquid substances of every kind, saline solutions, poisons, &c. It is now therefore obvious that, by the forcible entrance of arterial blood into the capillary vessels, the fluids contained in these—in other words, the soluble compounds produced by the transformation of the organized tissues—must, as above stated, be compelled to move towards the heart. These compounds cannot be employed for the reproduction of those tissues from which they are derived. They pass through the absorbent and lymphatic vessels into the veins, where their accumulation would speedily put a stop to the nutritive process, were it not that this accumulation is prevented by two contrivances adapted expressly to this purpose.

"A part of the venous blood, before reaching the heart, is made to pass through the liver: a part of the arterial blood, on the other hand, passes through the kidneys, which separate from it all substances incapable of contributing to nutrition. Of the newly produced compounds, some are collected in the urinary bladder; another part is separated by the liver in the form of *Bile*. Physiologists cannot entertain any doubt as to the origin of the constituents of the Urine and of the Bile. Even when all food is withheld, the secretion of bile takes place: in the bodies of those who have been starved to death, the gall-bladder is found distended and full of bile. * * * * * *

"It is obvious that the constituents of the urine, as well as the chief constituents of the bile, are products of the transformation of the blood and of the organized tissues. The elements of urea, uric acid, and bile, were previously constituent elements of the living tissues, which have lost the condition of life in the vital process by the action of external causes. We know with certainty, therefore, that some of the nitrogenized compounds, produced by the metamorphosis of organized tissues, after being separated from the arterial blood by means of the kidneys, are expelled from the body as utterly incapable of farther alteration.

"But another nitrogenized chief product, in which we find the sulphur of the transformed tissues, and which is peculiarly rich in carbon, returns, as bile, during the progress of digestion, into the system, in which it gradually disappears, partially or entirely. If we compare the composition of the bile with the nature and composition of the substances which are expelled through the intestinal canal, it evidently appears, that the combustible elements of the bile, without referring to the important part which that fluid plays in the process of digestion, ultimately leave the body in the shape of oxidized compounds, and are perfectly capable of being employed in respiration.

"The bile contains sulphur and is a compound of soda. The fæces of carnivorous animals, as of serpents and of dogs, fed with flesh and bones, contain a very small proportion of organic excretions, which promote the passage through the intestine of the substances not taken up during the digestive process. They consist chiefly of bone earth, and, when incinerated, yield only traces of soluble salts, among which, however, is not found carbonate of soda, which is left when bile is incinerated. It is perfectly certain the soda of the bile has again entered into the circulation. We find the soda and sulphur of the bile, the first in the form of a salt of soda, the latter in the form of a sulphate, in the urine.

"The peculiar property of the vessels of the intestinal canal, that of taking up and carrying into the blood soluble substances of every kind, provided these do not form an insoluble compound with the organic tissues of the vessels, is well-known. A solution of common salt (1 part to 80 of water) administered as an enema, disappears in the intestine just as pure water would do, without the proportion of salt in the fæcal evacuation an hour after being in the smallest degree increased. But the proportion of salt in the urine increases in a direct proportion. In like manner, ferrocyanide of potassium, or iodide of potassium, introduced in the same way into the rectum, very soon appears in the urine; and the disappearance of an enema of bile in the rectum, while the bile cannot be detected in the urine, proves, not only the passage or return of the bile into the blood, but also its employment in the respiratory process." P. 73–77.

*Employment of Alcohol in Respiration.*—The alcohol of fermented liquors plays the same part in the body of man as the non-nitrogenized constituents of his food; for although its elements do not of themselves possess at the temperature of the body the property of combining with oxygen, yet brought into contact with other bodies during their eremacausis or absorption of oxygen, which are always present in the body, it acquires the property in a higher degree than fat or other non-nitrogenized substance.

"When cod-liver oil is administered to persons accustomed to drink daily a certain quantity of wine, it often happens, that the inclination for wine is diminished, so that at last they can take no wine at all: obviously, because alcohol and fat oil in this case mutually impede the excretion of each other through the skin and lungs, since the body does not assimilate the fat. This may also possibly be the reason why most people find that they can take wine with animal food, but not with farinaceous or amylaceous food." P. 97.

Experiments prove that neither the urine nor the condensed fluid of expired air contain alcohol, whence it necessarily follows that its elements have been given out as oxidized products, its carbon as carbonic acid, and its hydrogen as water. When, however, the supply of oxygen is defective, the amount of the carbon of the alcohol being greater than that of the oxygen necessary for its conversion, "the alcohol must then pass off as such, or in the form of a lower stage of oxydation, as butyric or acetic acid: or else it must be discoverable in the body." The agency of alcohol as a respiratory element of food is illustrated by the following anecdote:—

"In England, servants receive daily a certain amount of Beer, or in the case of Total Abstinence, its equivalent in money. A friend informs me that in a certain household it was observed, that, from the day on which the servants ceased to receive beer from their master, the consumption of bread increased in a ratio corresponding to the diminution of beer; so that the beer was twice paid for, once in money, and the second time in the form of an equivalent of another kind of food, yielding the same amount of carbon and hydrogen." P. 98.

There is an interesting section upon the derivation of the various vegetable acids and starch, gum, &c. from the carbonic acid of the plant, and their reconversion into this in the animal; but, as abridgment is impossible, we content ourselves with a single extract.

*Respiration an indirect Combustion.*—"In vegetables, sugar has thus been formed from Carbonic Acid, by the separation of Oxygen, and by the introduction of Hydrogen in its place. In the animal organism, the process is reversed: the hydrogen, in the animal body, is removed and replaced by Oxygen; and in this way the Carbon again assumes its original form of combination.

"This is, generally speaking, the essential character of the process of eremacausis or respiration: it is an indirect process of combustion, going on at a low temperature, and with a limited supply of Oxygen. We are not acquainted with any case in which, under these circumstances, the Carbon of an organic substance combines directly with Oxygen to produce carbonic acid. No combustion of the carbon, in the proper sense of the word, takes place, but the Hydrogen of the compound is oxidized and separated as Water, while its equivalent of oxygen is taken up in its place. Should one of the intermediate compounds, which are formed by the gradual replacement of the Hydrogen by Oxygen, possess, in itself, an attraction for Oxygen, then for 1 eq. Hydrogen more than 1 eq. Oxygen is taken up. The development of heat in the respiratory process, therefore, depends not on the direct oxidation of the Carbon but on the conversion of the Hydrogen of the organic compound into water, and on the substitution of one or more equivalents of Oxygen for this Hydrogen." P. 110.

*The Elements of Respiration as Sources of Heat.*—The amount of heat produced in their oxidation varies much in these different constituents of food, being greater in proportion as they are rich in combustible or unoxidized Hydrogen—that being the substance during the combination of which with oxygen most heat is extricated. Estimating the heat produced during oxidation by the amount of oxygen required for converting different substances into carbonic acid and water, we find that 1℔ of Sugar of Milk combines with 187 volumes of oxygen; the same quantity of Cane Sugar with 196: of Starch with 207; of Alcohol with 362; and of Fat with 511 volumes. If 1 part of Sugar of Milk can keep up the normal temperature for 33 hours, the same quantity of Cane Sugar, Starch, Alcohol, and Fat, will maintain it for 35, 36, 65 and 87 hours respectively. "In order to keep the body at the same temperature during equal times, there are required, of Cane Sugar 100 parts, Grape Sugar 106, Starch 97·2, Alcohol 53·8, Fat 40·2, and Flesh 309·7. Pure flesh, therefore, possesses the smallest, and fat the greatest value as an Element of Respiration."

### *The Intestinal Canal an Organ of Secretion.*

"It is hardly to be doubted that when nitrogenized substances are to be found in the fæces, and their presence has been demonstrated by all the analyses hitherto made, these substances can only be, either products of the change of matter in the intestinal canal itself, or products of the general change of matter, which have not undergone the normal changes, and which are separated from the blood, in virtue of a power belonging to the intestinal canal, or to some portion of it. The apparent deficiency or absence of any structure in the canal, by means of which the secretory process is effected, is opposed to the opinion, that a true circulation, attended by a restoration of the disturbed equilibrium in the organism, goes on there : but the following considerations may perhaps serve as a support to that opinion.

"It is plain that the secretory effect of the canal, the amount of matters separated from the blood by its action, must stand in a definite ratio to the amount of oxygen taken up and consumed by the individual: or, what comes to the same thing, to the amount and composition of the food. Every change in the relative proportion of blood-constituents and non-nitrogenized elements of respiration in the food, must exert an influence on the quantity and composition of the fæces.

"If we assume, that the food contains a larger proportion of blood-constituents than is required for the supply of the waste of matter in the body, then the excess of these constituents must augment the mass of blood, or, if the animal possess the necessary assimilative power, the mass of flesh in the body. If the amount of oxygen taken up be exactly sufficient to convert into oxidized products in a given time the products of the change of matter present in the system, as well as the elements of respiration contained in the food, then the fæces must possess the normal composition and character. But if the amount of sugar or of fat introduced in the food be greater than the oxygen supplied in an equal time can completely convert into carbonic acid and water, then, in an animal possessed of the necessary power, a part of the sugar will be converted into fat; and this fat, along with the fat introduced in the food, will go to increase the quantity of fat in the body. If we now suppose the products of the change of matter and non-nitrogenised elements of respiration possess an equal attraction for the oxygen with which they combine in the organism, it is evident that the oxygen present must be divided between them. A certain portion will unite with the sugar, or with the elements of non-nitrogenized elements of respiration : another portion will combine with the elements of the nitrogenized products resulting from the change of matter. When the supply of oxygen is deficient, or, what comes to the same thing, when there is an excess of non-nitrogenized and nitrogenised elements of respiration (the latter being always viewed as the products of the change of matter), their normal conversion into oxidized compounds must necessarily appear impeded.

"Sugar, when oxygen is wanting, may pass into fat: but only a part of the products of the change of matter can be, under these circumstances, converted into the normal oxidized compounds. While, in the normal state of nutrition, of waste and restoration, and of the supply of oxygen, the nitrogen of the effete tissues takes the form of urea, and the sulphur of the bile that of sulphuric acid, which are discharged in the urine; when there is a deficiency of the oxygen necessary to the formation of these products, uric acid, a compound much richer in carbon than urea, will be formed, a part of the sulphur will appear in the urine as cystine (cystic oxide), or in some other form; and the excess of the products of the change of matter which has not undergone these changes, must either remain in the blood, or it must, as we know of no other exit for it, be evacuated by the intestinal canal." P. 144–146.

It will be probably more convenient to notice Dr. Thomson's interesting little work upon "The Food of Animals," before giving any further account of the one we are occupied with. It consists in the description of a series of well-contrived experiments upon the comparative value of various articles of diet in furnishing supplies of milk to cows, and is consequently of more immediate interest to the agriculturist than to the medical reader. Still, being the work of an accomplished chemist and physician, it contains several incidental observations of value to the practitioner, to some of which we will allude. Upon the whole, the book is an admirable exemplification of the great practical advantages derivable from pursuing the track of investigation so lucidly indicated by Liebig.

*Influence of Variety of Food on Milk, and on Man.*—Several series of experiments proved in a decided manner that, upon a change of food, the amount of milk produced became increased, diminishing again after the same diet had been continued for some days.

"That a change of diet is necessary for animals which are kept in a confined condition, is proved by the tables previously given, in a striking manner, and the results now obtained amply sustain the idea supported by me some time ago in reference to the dietary of human beings shut up in poor-houses and places of confinement. It was then argued that 'in order to retain the human constitution in a healthy condition, variety of food should be properly attended to,' and different species of diet were suggested as well calculated to supply a series of dishes to the poor. In the Asylum for the Houseless, and in the House of Refuge at Glasgow, the recommendations were followed out; and, according to the report of the treasurer, 'the dinner meals being varied two or three times a week, the change in the dietary routine is much relished by the inmates, and may have had some effect in the greater degree of health which has been evident among them of late.' (*Proceedings of Phil. Soc. of Glasgow,* Vol. 1, p. 40). The analogy subsisting between the physical nature of human beings and of many of our domestic animals would lead us to the conclusion, upon physiological grounds, that the dietary should be conducted upon precisely the same principles. To prove this by exact experiment is a point, it will be admitted, of considerable importance to the agriculturist, although it may have been, as might be expected, surmised by many intelligent observers. Not only, however, is a *variety* of food requisite for an animal in an artificial state, it is found also to be beneficial to one in a condition more akin to that of nature. For it is upon this principle we are able to account for the superior influence of old natural pastures, which consist of a variety of grasses and other plants, over those pastures which are formed of only one grass, in the production of fat cattle and good milk cows. To any one who considers with attention the experiments which have been detailed, there cannot remain a doubt in the mind that cattle, and especially milk cows, in a state of confinement, would be benefited by a very frequent and entire change in their food. It might not be too much to say that a daily modification in the dietary of such animals would be a sound scientific prescription. In considering the case of the white cow, we find that a change from barley to barley and molasses increased the milk in three days from 21lb. 6 oz. to 23 lb. 7 oz.; on changing from malt to barley it increased from 19 lb. 10 oz. to 20 lbs. 11 oz. on the first day: from barley to barley and linseed, it increased from 21 lb. 2 oz. to 23 lb. 12 oz. on the sixth day: from barley and linseed to beans, it increased on the first day from 21 lbs. 13 oz. to 23 lbs. 14 oz." P. 147.

We consider the foregoing extract of high importance, in pointing to an omission entailing much needless suffering. Many poor-law and prison dietaries are constructed with sufficient liberality as regards quantity, but in utter neglect of the no less important item variety. The same unvaried routine is the doom of the unfortunate inmates, the severity of which may be judged of by the palling effect which the too frequent repetition of even the most excellent dishes exerts upon the palates of persons not condemned to the miseries of inaction. Common humanity demands that this matter shall be looked into, especially as the rectification of the error involves no additional expense, inasmuch as Dr. Thomson suggests some cheap varieties, and others might be easily added.

Dr. Thomson suggests *Calorifiant* or *Heat-producing* as a more suitable term for designating the Elements of the food termed *Respiratory* by Liebig, and the following are some of his observations upon the proportion which these hold to the Nitrogenized or Nutritive Elements.

"Milk, the food of the infant mammalia, contains one part of nutritive to two parts of calorifiant constituents, and in the young state of an animal the nutritive part of the food not only supplies the place of the metamorphosed solids, but an additional amount of it is required to increase the bulk of the individual; and, as we have already stated that animal heat is generated by the change or degradation of the fibrinous tissues, it is obvious that, in the nourishment of infant life, there is a supply of heat, from the casein, vastly superior to that afforded by fibrin supplied to full-grown animals, because the amount taken in proportion to the quantity of calorifiant matter is much greater. If we refer, again, to the food which is generally employed by the inhabitants of this country, wheat and barley, we find, by a mean of experiments afterwards to be detailed, that the average amount of albuminous matter present in them is 11 per cent., while the quantity of starch and sugar existing in those substances may vary from 70 to 80 per cent.: thus affording the proportion of nutritive to calorifiant food as 1 to 7 and upwards. Such food, it may be inferred, is fitted for the consumption of an animal which is not subjected to much exercise of the muscular system, and may be viewed as the limit of excess of the calorifiant, over the nutritive constituents of food. As the demands upon the muscular part of the frame become more urgent, the proportion of the azotized constituents should be increased, and this may be extended until we arrive at the point where the fibrinous matter is equal to the half of the calorifiant, which is probably, in a perfectly normal physiology, the greatest relative proportion of nutritive material admissible." P. 165.

Dr. Thomson observes that, in order to vary the nature of the food according to the rest or activity of the animal, tables of its composition are essential, and he presents a short one founded upon his own experiments. From this, it appears that the relative proportions of nutritive to calorifiant matter in various articles of diet are as follow:—Milk 1 to 2; Beans 1 to 2½; Oatmeal 1 to 5; Barley and Semolina 1 to 7; English Wheat Flour 1 to 8; Potatoes 1 to 9; Rice 1 to 10; Turnips 1 to 11; Arrow-root, Tapioca, and Sago 1 to 26; Starch 1 to 40. From the accompanying remarks we select the following interesting ones on the *Food of Children.*

"Hence we learn that milk, in some form or other, is the true food of children, and that the use of arrow-root, or any members of the starch class, where the relation of the nutritive to the calorifiant matter is as 1 to 26, instead of being as 1 to 2, by an animal placed in the circumstances of a human infant, is opposed to the principles unfolded by the preceding table. In making this statement, I find that there are certain misapprehensions into which medical men are apt to be led at the first view of the subject. To render it clearer, let us recal to mind what the arrow-root class of diet consists of. Arrow-root and Tapioca are prepared by washing the roots of certain plants until all the matter soluble in water is removed. Now, as albumen is soluble in water, this form of nutritive matter must in a great measure be washed away: under this aspect we might view the original root before it was subjected to the washing process, to approximate in its composition to that of flour. If the latter substance were washed by repeated additions of water the nitrogenous or nutritive ingredients would be separated from the starchy or calorifiant elements, being partly soluble in water, and partly mechanically removed. Arrow-root, therefore, may be considered as flour deprived as much as possible of its nutritive matter. When we administer arrow-root to a child it is equivalent to washing all the nutritive matter out of bread, flour, or oatmeal, and supplying it with starch: or it is the same thing approximatively as if we gave it starch: and this is in fact what is done, when children are fed upon what is sold in the shops under the title of '*Farinaceous Food,*' empirical preparations of which no one can understand the composition without analysis. Of the bad effects produced in children by the use of these most exceptionable mixtures, I have had ample opportunities of forming an opinion, and I am inclined to infer that many of the irregularities of the bowels, the production of wind, &c. in children, are often attributable to the use of such unnatural species of food. It should be remembered that all starchy food deprived of nutritive matter is of artificial production, and scarcely, if ever, exists in nature in an isolated form. The administration of the arrow-root class is therefore only admissible when a sufficient amount of nutritive matter has previously been introduced into the digestive organs, or when it is inadvisable to supply nutrition to the system, as in cases of inflammatory action. In such cases, the animal heat must be kept up, and for this purpose calorifiant food alone is necessary. This treatment is equivalent to removing blood from the system, since the waste of the fibrinous tissues goes on, while an adequate reparation is not sustained by the introduction of nutritive food. A certain amount of muscular sustentation is still, however, effected by the arrow-root diet; since, according to the preceding tables, it contains about one-third as much nutritive matter as some wheat flours. The extensive use of *Oatmeal,* which is attended with such wholesome consequences among the children of all ranks in Scotland, is, however, an important fact, deserving serious consideration: and, it appears to me, is strongly corroborative of the principles which I have endeavoured to lay down in the preceding pages." P. 169–171.

Dr. Thomson offers several useful observations upon the making of breads by the admixture of various flours, and is, we are glad to find, an advocate for the manufacture of this article independently of fermentation. If the experiments which have been recently so extensively undertaken prove successful, the boon conferred upon humanity will not be inconsiderable, when we consider the facility with which fresh-bread may be made on ship-board, and the emancipation of that numerous class of persons the journeymen bakers from their destructive nocturnal toils. We believe the experience of most persons who have tried it is in favour of the comparatively greater wholesomeness of unfermented bread, and Dr. Thomson, as the result of his investigations, finds its production more economical than is that of the fermented:—a sack of flour yielding 107 loaves of unfermented, and but 100 of fermented bread; 6½ per cent. being lost by the ordinary process of baking. He furnishes the following formula for *making unfermented bread.*

"Take of Flour 4 pounds, Sesquicarbonate of Soda 320 grains, Hydrochloric Acid 6½ fluid-drachms, Common Salt 300 grains, Water 35 ozs. by measure. The Soda is first mixed with the Flour very intimately. The Salt is dissolved in the water, and added to the Acid. The whole is then rapidly mixed as in common baking. The bread may be either baked in tins or formed like cottage loaves, and should be kept from one to two hours in the oven. Should it prove yellow, it is a proof that the soda has been in excess, and indicates the propriety of adding a small additional portion of acid; the acid varying somewhat in strength." P. 185.

We now proceed to the consideration of Baron Liebig's observations upon "The Mutual Relations of Chemistry and Physics to Physiology and Pathology," as contained in his new Chapter upon the mode in which the investigation of the Metamorphoses of Animal Tissues should be pursued. It is an excellent chapter, full of sound philosophy, and we are much pleased to find it's author acknowledging himself so much indebted to that admirable work, "*Mill's System of Logic,*" during its composition. "Indeed, he feels that he can claim no other merit than that of having applied to some special cases, and carried out, further than had been previously done, those principles of research in natural science which have been laid down by that distinguished philosopher." Did works of this stamp more frequently form a portion of the preliminary studies and future references of the members of our profession, we should be spared many a crude theory and disjointed hypothesis, and should seldomer see them entangled in the mazes of sophistical reasoning and profitless speculation.

To proceed: Although in certain of the natural sciences the mutual relations of their phenomena have been observed with sufficient minuteness and accuracy to permit, by the special laws thus accumulated, the safe deduction of general laws, competent to the explanation of new phenomena and observations; such a point has not been reached as respects Physiology. To render it a deductive science, however, precisely the same procedures are requisite, and its progresss is retarded by the same obstacles which have impeded that of other branches of science. Of these the difficulty of overcoming preconceived opinions by reason of some imperfection of information defying comprehension is the greatest; and thus many now well-established and easily understood facts of science positively defied at one time the comprehension of the most sagacious minds.

"We thus perceive that 'the comprehensible' has nothing whatever to do with the phenomenon. It depends upon the state of the development of the intellect. When, to the observer, the connecting link is wanting, which attaches a fact to the ordinary course of thought, then the fact, to his view, is destitute of truth or comprehensibility. This is one of the greatest obstacles which impedes the application of Chemistry to Physiology, or the simple study of chemical discoveries on the part of many physiologists. To this must be added, in Pathology, the holding for true of observations, the accuracy of which has no other support than this, that they have been held as true for a thousand years. If, in these branches of knowledge, the methods of proof and investigation be not changed, there is no hope, with all her progress, Chemistry can ever be capable of yielding essential advantages to Physiology and Pathology; and yet it is impossible that these branches can ever acquire a scientific foundation, without the aid of Chemistry and Physic. Everyone feels the necessity: it is only concerning the mode of application of these sciences that men are not agreed." P. 162.

One great cause of the modern progress of Chemistry consists in the acknowledgment of a plurality of causes of its various conditions, in contradistinction to the essential properties of the older chemists; but too many physiologists and pathologists of the present day comprehend under the terms Nervous Force, Vegetation, Irritation, &c. so many independent existences analogous to the essences of the phlogisticians. Thus the terms endosmose and exosmose are regarded, not merely as expressive of one of the modes of filtration, but as denoting essential properties. Although it should be a primary object to distinguish the various conditions upon which the phenomena of vitality depend, and this principle is often applied in modern physiological investigations, yet M. Liebig, in a critical commentary upon the doctrines of Irritation, delivered by Professor Henle in his Pathology, shows that, even at the present time, very opposite conditions are often confounded together under some ill-defined or paraphrasistical term, false analogies and comparisons being also frequently resorted to. We have not space to follow him through this exposition, but may quote a short passage.

"A rude image of the organism, in many of its relations, may be found in the great sea-going steam-vessels. These consume, at each moment of their voyage, oxygen and fuel, which are given out in the form of carbonic acid, water, and soot or smoke. In them, exists a source of heat and a source of power which produces motion, or prepares the food of the crew; and when a sail is torn, there is a man at hand to repair it: a leak is stopped by the carpenter: blacksmiths and other hands are active to preserve the ship in her original state and in motion. So, also, in the living body, there are smiths and carpenters at work, and the problem is, to acquire a knowledge of them, and of their mutual relations." P. 177.

An exercised condition of the perceptive powers is an essential condition for accurate *observation;* and no merely theoretical views in Chemistry or Physics will be received at the hands of one who has not given, by his prior practical investigations, assurance of his powers of correct appreciation. "It required a Berzelius, with his acute perceptive powers, to save from oblivion the notions of Richter concerning chemical proportions, to recognize their interior truth, and the existence of an universal law of combination under a mass of false facts, of which a single example—that of the carbonate of alumina, employed as the starting-point of the first table of equivalents, *which salt does not exist*, was sufficient to destroy all belief in the more accurate remaining facts." We cannot do better than quote the following excellent remarks upon *erroneous observation.*

"From the point of view of true natural philosophy every erroneous mode of contemplation and interpretation depends on the want of correct observations and on the false notion entertained of the nature of an observation. It further depends on this, that we regard the constant association of two things, or the constant occurrence simultaneously of two phenomena, as a necessary connection, and consider them as mutually determining one another. In nature, a number of phenomena appear together, one of which is not perceived when the other is wanting, but there are numberless others which occur together, without the remotest connection. The supposition of a relation thus fallacious, or a false *nexus causalis* proceeds in all cases from a false method of observation. So, also, the association of two phenomena, which are only analogous in one solitary relation, is always the result of imperfect observation.

"To see, to perceive by means of our senses, is one condition of observing: but seeing and perception do not characterize true observation. The problem to be solved by the observer, is, not merely to see the thing, but also the parts of which it consists. A good observer must notice and seek to convince himself, in what connection the facts stand to each other and to the whole." P. 183.

Several examples of erroneous observation are adduced; but the one commented upon at greatest length, is that furnished by the *parasitic theory of contagion.* In opposition to this, the author first states the facts favorable to the chemical theory of contagion, giving a luminous account of the origin and bearings of *ferments* and *putrefaction.* To the question of whether the state of transformation or putrefaction may be propagated to the living organism? he believes facts answer affirmatively. Among these are the effects of wounds received during dissection, and the symptoms induced by the ingestion of food in a state of decomposition, as German sausages, &c. The products of disease so induced are neither more or less than portions of the constituents of the frame in a state of change in form or composition, and by means of such matters, so long as the state of decomposition continues, may the disease be propagated to others. Moreover, antiseptic substances powerful in checking putrefaction are those best adapted to destroy the communicability of contagions; while ammonia, the common product of putrefaction, is abundantly discharged from the system in typhus. Finally, the origin of epidemic and miasmatic disease in localities wherein decomposition of organic matters is going on is familiar to all observers.

"Hence, according to all the rules of scientific investigation, the conclusion is fully justified, that in all cases where a process of putrefaction precedes the occurrence of a disease, or where the disease can be propagated by solid, liquid, or aeriform products of disease, and where no nearer cause of this disease can be discovered, the substances in a state of decomposition or transformation must be regarded as being, in consequence of that state, the proximate causes of the disease. The condition which determines, in a second individual, his liability to the contagion, is the presence, in his body of a substance, which, by itself, or by means of the vital force acting in the organism, offers no resistance to the cause of change in form and composition operating on it. If this substance be a necessary constituent of the body, then the disease must be communicable to all persons; if it be an accidental constituent, then only those persons will be attacked by the disease, in whom it is present in the proper quantity, and of the proper composition. The course of the disease is the destruction and removal of this substance; it is the establishment of an equilibrium between the cause acting in the organism, which determines the normal performance of its functions, and a foreign power, by whose influence these functions are altered." P. 206.

The author is quite at a loss to imagine how the *parasitic theory* of contagion can be preferred by any enquirer to the one above stated. It is based upon two facts, the propagation of scabies, and the occurrence of the disease called muscardine in the silk-worm. That the contagion of *scabies* is an animal is obvious, and its communication by this means requires no theory to explain it: but when we come to inquire respecting all other contagious diseases, as small-pox, &c., we find the most careful observation has never detected any organized beings capable of propagating the contagion. A disease like scabies, therefore, which is caused by the development of a parasite in the body, must not be confounded with others in which such condition is absent; whatever resemblance other apparent phenomena may present. " The opinion that infection in contagious diseases is caused by living beings, and that scabies is to be regarded as the type of contagious diseases, has been chiefly founded on the principle that like effects imply like causes. This is the very principle which for centuries impeded the progress of natural science, and which, even in the present day, leads to so many errors."

Of the propagation of purely miasmatic disease nothing is known, and neither the chemical or parasitic theory attempts their explanation; but the latter points to the muscardine as the type of the contagious miasmatic diseases which are induced by matters derived from the air as well as those derived from the system. The muscardine is a disease of the silk-worm caterpillar induced by a fungus, which grows at the expense of the body of the insect, and just as various other parasites of the vegetable and animal world, induces the death of the animal it infests; but there is no connexion between this and the origin of contagious miasmatic disease.

The parasitic theory has been based upon a fallacious explanation of fermentation and putrefaction, according to which these processes are caused by the presence of Infusoria and Fungi, a breeding-place for which is thus formed in every putrefying organic substance. The germs of these beings fill the atmosphere and become the causes of disease. The connexion existing between putrefaction and contagion is here acknowledged, though faultily explained. In fact, fungi and infusoria are, upon the extinction of the vital principle, subjected to the same reduction from more to less complex combinations as are all other varieties of organized matter, during putrefaction. It is true that fungi and infusoria are abundantly found in fermenting and putrefying bodies; but they are the accompaniments, not the causes, of these states. Generally they only appear when putrefaction has already begun, or is completed, and the process of decay commenced, dependent as they are for subsistence upon the organic atoms which have been set free by these processes. These bodies, indeed, by assisting in the resolution of the products of the organism into carbonic acid and carbonate of ammonia, the object of the putrefactive process, remarkably accelerate its progress, and are in fact antagonists to its continuance, and the enemies and destroyers of contagion and miasms. Indeed, during the active condition of the green and red infusoria they become sources of the purest oxygen gas.

We regret that our limits will not permit our following the author through his exposure of other fallacies, and his observations upon the dependence which exists between the physical properties of the elements and those of the organic compounds. After adducing numerous examples of such mutual dependence in purely chemical compounds, he proceeds:

" It is their method of investigation which has led many physiologists and pathologists to regard the vital properties in some measure as exceptions to a great law of Nature. How else can it be explained that they do not consider the number and grouping of the elements of which the organic tissues are composed, as a physiological property, which must serve as an altogether indispensable means of acquiring a knowledge of vital phenomena? How can it be explained that they do not take into account, in curing and removing diseased conditions, the elementary composition of their remedies, and the properties thereon depending, by which their effects are produced? A mere knowledge of formula is evidently not sufficient, but it is absolutely necessary to discover the laws which regulate the relations of the composition and form of the food or of the secreta to the nutritive process, and of the composition of remedies to the effects which they produce on the organism." P. 248.

The present advanced state of physiology would have been impossible without that complete knowledge of structure which anatomy imparts: but anatomy alone is not sufficient to ensure future progress. If it were, chemistry could lend no helping hand, inasmuch as its office is not "to discover the form, but to determine the relation between the form and the elements, with their arrangement, by which that form is produced."

"If anatomical knowledge is to serve for the resolution of a physiological question, then, of necessity, something more must be called in, and the most obvious is the matter of which the organized form consists, the forces and properties which belong to it, in addition to the vital properties, and a knowledge of the origin of this matter, and of the changes which it undergoes in order to acquire vital properties. Finally, it is indispensable to know the relations in which all the constituents of the organism, the fluid as well as the solid, altogether independently of the form, stand to each other. To many physiologists, chemistry alone appears to have been enriched by all which chemistry has discovered, with reference to these highly important questions, although in chemistry, all these results occupy a rank quite as subordinate as that attained by the analyses of minerals and of mineral waters.

"Another fundamental error, committed by other physiologists, is this, that they suppose the chemical and physical forces alone, or in combination with anatomy, sufficient to explain the phenomena of vitality. It is indeed difficult to understand, how the chemist, intimately acquainted with chemical forces, recognises in the living body the existence of new laws, of new causes, while the physiologist, little or not at all familiar with the knowledge of the action and nature of chemical and physical forces, is ready to explain the same processes, with the aid of the laws of inorganic nature alone.

"The last-mentioned view is, in reality, the extreme consequence of a re-action against a previous one. In the age, not yet long past, of metaphysical physiology, every thing was explained by the vital force. The re-action rejects the vital force, and believes in the possibility of reducing all vital processes to physical and chemical causes. 'In the living body prevail,' thus men spoke forty years ago, 'other laws than those of inorganic nature.' Many physiologists of the present day, on the other hand, regard them as of the same kind. That which, in both views, is profitless for us, is that, neither formerly nor at this time, have men endeavoured to determine or discover the differences in the effects of the vital force, and those of the inorganic forces, and their likeness or unlikeness. The conclusions arrived at were not founded on a knowledge of the likeness or unlikeness of their mutual relations, but on ignorance of these things.

"The same physiologists, who regard the vital processes as the effects of inorganic forces alone, forget entirely, that by the expression 'chemical forces' is meant nothing else than the *quantitative* in the different vital phenomena, and the *qualities* determined by these quantities. From the false notion which has been formed of the influence of chemistry on the explanation of the vital phenomena, it has happened that, on the one hand, this influence has been estimated too low, and that, on the other, the expectations and demands on it have been raised too high." P. 252.

